# Evolution of Antibacterial Drug Screening Methods: Current Prospects for Mycobacteria

**DOI:** 10.3390/microorganisms9122562

**Published:** 2021-12-10

**Authors:** Clara M. Bento, Maria Salomé Gomes, Tânia Silva

**Affiliations:** 1 I3S–Instituto de Investigação e Inovação em Saúde, Universidade do Porto, 4200-135 Porto, Portugal; clara.bento@i3s.up.pt (C.M.B.); sgomes@ibmc.up.pt (M.S.G.); 2 IBMC–Instituto de Biologia Molecular e Celular, Universidade do Porto, 4200-135 Porto, Portugal; 3 Programa Doutoral em Biologia Molecular e Celular (MCBiology), Instituto de Ciências Biomédicas Abel Salazar, Universidade do Porto, 4050-313 Porto, Portugal; 4 ICBAS–Instituto de Ciências Biomédicas Abel Salazar, Universidade do Porto, 4050-313 Porto, Portugal

**Keywords:** drug susceptibility testing, antimicrobial activity, antimicrobials, drug screening, high-throughput, *Mycobacterium*, reporter strains, granulomas, biofilms, organoids

## Abstract

The increasing resistance of infectious agents to available drugs urges the continuous and rapid development of new and more efficient treatment options. This process, in turn, requires accurate and high-throughput techniques for antimicrobials’ testing. Conventional methods of drug susceptibility testing (DST) are reliable and standardized by competent entities and have been thoroughly applied to a wide range of microorganisms. However, they require much manual work and time, especially in the case of slow-growing organisms, such as mycobacteria. Aiming at a better prediction of the clinical efficacy of new drugs, in vitro infection models have evolved to closely mimic the environment that microorganisms experience inside the host. Automated methods allow in vitro DST on a big scale, and they can integrate models that recreate the interactions that the bacteria establish with host cells in vivo. Nonetheless, they are expensive and require a high level of expertise, which makes them still not applicable to routine laboratory work. In this review, we discuss conventional DST methods and how they should be used as a first screen to select active compounds. We also highlight their limitations and how they can be overcome by more complex and sophisticated in vitro models that reflect the dynamics present in the host during infection. Special attention is given to mycobacteria, which are simultaneously difficult to treat and especially challenging to study in the context of DST.

## 1. Introduction

In a time when infectious diseases have taken the spotlight, it is relevant to question and discuss the methodologies behind the development of new anti-infectious agents. Regarding bacterial infections, the rapid emergence and spread of antibiotic resistance urges the discovery of new molecules that need to be screened rapidly and efficiently. The first methods developed to assess the efficiency of antibiotics are simple and globally reliable, but usually of low-throughput. They are often limited to qualitative results and do not reflect, in its complexity, the drug’s dynamics inside the infected individual. In view of these limitations, new methodologies had to be developed for DST. Recent technological advances allowed the development of new approaches that are nowadays indispensable to develop new drugs against prevalent pathogens. Among these, mycobacteria are particularly challenging. The *Mycobacterium* genus comprises the current world’s deadliest bacterial agent, *Mycobacterium tuberculosis*, but also *M. leprae*, the agent of leprosy, and several species of non-tuberculous mycobacteria (NTM), including *M. avium*, the most-prevalent worldwide, and *M. abscessus*, the most difficult to treat [1,2,3]. According to their growth rate on agar, mycobacteria can be classified as rapidly growing mycobacteria (RGM), such as *M. abscessus* or *M. chelonae*, which form visible colonies on agar in less than 7 days, or as slowly growing mycobacteria (SGM), such as those in the *M. avium* complex or *M. tuberculosis*, which take 7 or more days to form visible colonies on agar [3]. In the host, mycobacteria proliferate inside phagocytic cells, such as macrophages, multiplying inside small vacuoles [4]. The most common clinical presentation of mycobacterial infections is a pulmonary disease, but these pathogens can also cause skin and soft tissue infections, lymphatic, and disseminated disease [5]. Current treatment regimens include at least three drugs administered for 6 to 20 months, with serious side effects being common. Understandably, patients’ compliance is low, resulting in treatment failure and increased selection of multi-drug resistant strains. Thus, there is a need for new treatments [6] and, consequently, new methods to test them. This article reviews DST methods, from the simplest, such as broth dilution and agar diffusion, to the most complex and closest to in vivo, such as organoids and organs-on-a-chip. Their advantages and limitations are discussed, as well as their applications to different bacterial species, with a special focus on mycobacteria.

## 2. Gold-Standard Methods

### 2.1. Dilution Methods

The reference methods for DST are dilution methods. There are different committees and organizations that publish international standards and guidelines for DST testing, such as the Clinical and Laboratory Standards Institute (CLSI) [7], the European Committee on Antimicrobial Susceptibility Testing (EUCAST) [8], the European Centre for Disease Prevention (ECDC) [9], and the Centers for Disease Control and Prevention in the United States (CDC). Both broth and agar dilution methods are used to determine the drug’s minimum inhibitory concentration (MIC), which is the lowest concentration of a compound that inhibits the growth of a microorganism [10]. Broth dilution can be performed on tubes (macrodilution) or in microtiter plates (microdilution), in which two-fold serial dilutions of the testing compound are incubated with a standardized concentration of bacterial suspension. After incubation, which varies from 24 h to several days, according to the bacterial species and strain, the MIC is indicated by the first tube or well that has no visible growth [11,12,13]. The detection of turbidity can be tricky and have different interpretations by different observers. Furthermore, a series of 1:2 dilutions does not give short enough intervals of drug concentrations to determine with certainty the MIC of that drug. This is only correct if assumed that there is an “all or nothing” point for the susceptibility of a microorganism to a drug, which is usually not the case. To facilitate the reading of the MIC, or to draw a dose-response curve, cell metabolic activity can be measured by colorimetric methods, resorting to dyes such as tetrazolium salts (MTT or XTT) or resazurin [11]. Resazurin is a non-fluorescent compound that is reduced to fluorescent resorufin by metabolically active cells. Plotting the measured fluorescence as a function of drug concentration is a lot more informative than the MIC determined by turbidity observation. With that dose-response curve, it is possible to calculate the drug’s concentration that inhibits 50% or 90% of bacterial growth, called IC_50_ and IC_90_, respectively. Of note, several studies present results in the form of MIC_50_ or MIC_90_, which is not adequate as they do not represent minimal concentrations. Broth dilution also enables the determination of the minimum bactericidal concentration (MBC), the lowest concentration of the compound that kills 99.9% of the microorganisms [14]. For that, the content of a dilution tube or well with no visible microbial growth is plated on an antibiotic-free solid medium and analysed for growth after incubation [11]. This allows researchers to distinguish between bacteriostatic (MIC << MBC) and bactericidal (MIC ≈ MBC) compounds. A variation of the broth microdilution method, the checkerboard dilution test, allows assessment of the effect of drug combinations against the same microbial strain or to isolate and identify synergism [15]. This is particularly relevant in the case of mycobacteria since they are always treated with more than one antibiotic. In a microtiter tray, the bacterial suspension is in contact with dilutions four to five-fold below and over the MIC of one drug on the x-axis, and a second drug on the y-axis. The drugs’ synergism or antagonism is given by the fractional inhibitory concentration (FIC) index, which relates the drugs’ concentrations that inhibit the growth when used in combination with the MIC of each drug when used alone. A synergy is said to exist when FIC < 0.5 [15,16]

(1) $$\mathrm{FIC}\;\mathrm{index}=\left(\frac{\mathrm{MIC}\;A\;\mathrm{in}\;\mathrm{combination}}{\mathrm{MIC}\;A\;\mathrm{alone}}\right)+\left(\frac{\mathrm{MIC}\;B\;\mathrm{in}\;\mathrm{combination}}{\mathrm{MIC}\;B\;\mathrm{alone}}\right)$$

where A and B are two different drugs. This type of assay was used, for example, to identify synergistic drug combinations, such as spectinomycin and chlorpromazine, able to restore therapeutic efficacy against *M. tuberculosis* strains resistant to one of the tested drugs [17]. It is also useful to understand if emerging compounds, such as antibacterial peptides, besides being active against a bacterial strain, also have their activity enhanced by combination with conventional drugs [18].

**Figure 1:**
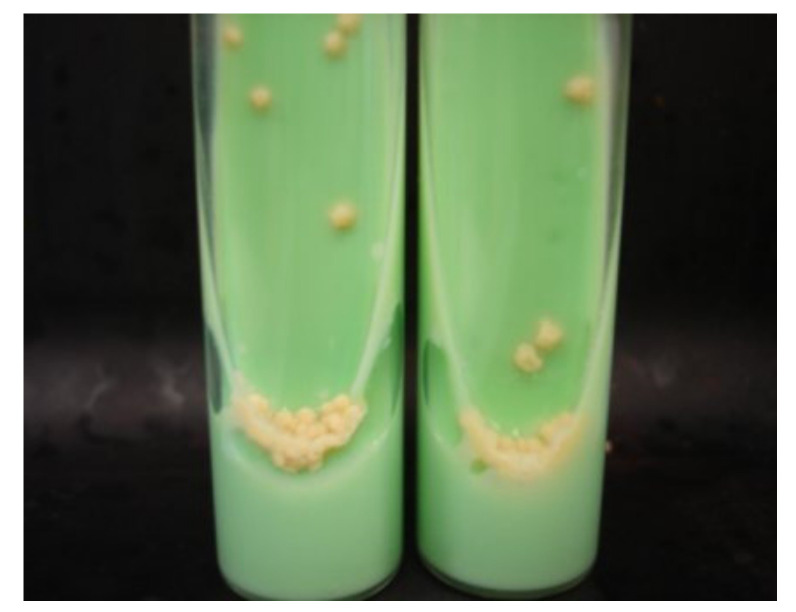
*M. tuberculosis* growing on Löwenstein–Jensen medium in slant tubes (from [22] under the terms of a CC BY 4.0 license: https://creativecommons.org/licenses/by/4.0/) (accessed on 9 December2021).

Agar dilution follows the same principle as the broth dilution assay. However, it has the advantages of overcoming problems with colored compounds that interfere with growth detection on a liquid medium and allow the prompt identification of contaminant colonies [11,19]. The bacterial load, after contact with different antibiotic concentrations, is determined by counting colonies and calculating colony-forming units (CFUs) [12]. The addition to the agar medium of chromogenic substrates that target specific microbial enzymes may be used to differentiate between bacterial species and strains with different antibiotic susceptibilities in a mixed culture and facilitate colony counting [20]. One of the methods available for DST on mycobacteria is the proportion method on Löwenstein–Jensen (LJ) medium [9]. This solid culture medium does not contain agar but an egg suspension, which, upon heating, coagulates, making a solid surface for mycobacterial colony formation (Figure 1). Appropriate dilutions of a bacterial suspension (that are going to yield a countable number of colonies) are seeded in the LJ medium with or without different concentrations of drugs in screw-capped tubes. The tubes are incubated in a slanted position until colonies appear on the surface of the slant and a proportion of resistant bacteria is calculated. A strain is classified as resistant to an antibiotic if it grows more than 1% on the drug-containing media, compared with the growth on drug-free control media [9,21].

Besides being highly time- and resources-consuming, the dilution methods do not provide prompt results, even for rapidly-growing bacteria. For slowly-growing NTM, the activity of a compound takes at least one week to assess, while a minimum of three weeks is needed for the *M. tuberculosis* complex. For broth dilution methods, the use of colorimetric reagents such as resazurin or MTT, as mentioned above, allows having a quicker readout, which does not happen for agar dilution methods.

### 2.2. Agar Diffusion Methods

Methods based on agar diffusion, either using impregnated filter paper discs as the Kirby–Bauer method, strips as the epsilometric test (E-test), or direct placing of the compound on a hole in the culture medium, are widely used to evaluate drug resistance in rapidly growing aerobic microorganisms [11,12]. The contact between the compound and the microorganisms in an inoculated agar plate will create an inhibition zone that can be measured and compared with standard values, indicating if the strain is resistant, intermediate, or susceptible to the antibiotic [11,12,13]. However, several factors can affect these diffusion methods, such as the culture media, the potency of the discs, the molecular weight and structure of the compound, and the type of microorganism [13,19]. Indeed, the growth rate of the microorganisms influences the size of the inhibitory zone. By the time slow growers reach a proper bacterial density, the antibiotic concentration around the disk may be below the predicted, resulting in smaller inhibition zones, which hampers the proper interpretation of the results [19]. Bacteria that grow very slowly, such as some mycobacteria, are even excluded from the CLSI guidelines for disk diffusion methods [23]. Instead, DST by dilution methods is recommended. For rapid-growing mycobacteria, such as *M. fortuitum* or *M. chelonae*, disk diffusion assays were performed [24,25] but with poor correlation with broth dilution methods for several antibiotics. The disk diffusion method has the disadvantages of not providing the MIC of the compound or discriminating between a bacteriostatic or bactericidal effect [11,12]. The former is overcome by the Epsilometer test (E-test), commercially Etest^®^ (bioMérieux), in which a strip with an increasing concentration of antibiotic is placed on an inoculated agar plate. The MIC is read where the formed inhibitory ellipse intersects the antibiotic concentration written on the strip [11,12]. This test also allows studying the synergistic effect between two compounds against a certain microorganism, by, for example, placing the two strips in a cross-formation at the individual MIC values on the agar plate. The resulting MICs allow calculating the FIC index [11]. Although being routinely used with a large range of bacteria [12], it has been shown that the MICs determined by the E-test can be slightly discrepant from those obtained by the CLSI reference dilution methods, for example, with methicillin-resistant *Staphylococcus aureus* isolates [26]. Concerning this limitation, studies with mycobacteria show inconsistent conclusions. For *M. tuberculosis*, Joloba et al. [27], Hazbón et al. [28], and Akcali et al. [29] showed that the agreement between E-test and a reference agar method is above 95% for the detection of resistance to different first-line drugs. On the other hand, Hausdorfer et al. [30] found false resistance in eight *M. tuberculosis* strains by E-test, using a radiometric system as a reference method. Verma et al. [31] found an overall agreement of only 48.6% between E-test and a reference dilution method. Studies with non-tuberculous mycobacteria such as *M. marinum* [32] and *M. kansasii* [33] show a high correlation between E-test and a reference dilution method.

## 3. Microcalorimetry

**Figure 2:**
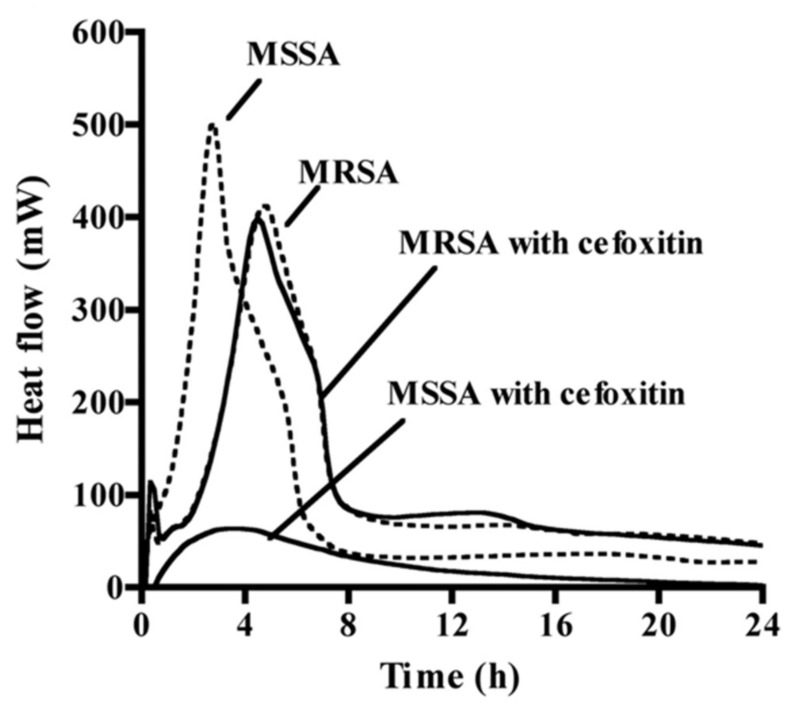
The different heat profiles obtained by microcalorimetry of methicillin-susceptible *S. aureus* (MSSA) and methicillin-resistant *S. aureus* (MRSA) in the presence (solid lines) or not (dashed lines) of the antibiotic cefoxitin (from [35], adapted with the permission of the American Society for Microbiology, 2021, conveyed through Copyright Clearance Center, Inc., Danvers, MA, USA).

Bacterial susceptibility can be assessed by microcalorimetry, a method that measures in real time the production of heat associated with bacteria’s growth and metabolism [12]. Sealed ampoules with culture medium, bacteria, and an antibiotic are placed inside a multichannel microcalorimetry instrument that measures the heat flow in each ampoule continuously, allowing to plot growth and dose-response curves [34]. This method can clearly distinguish the bacteria’s susceptibility and resistance to antibiotics, as observed by the different heat profiles between methicillin-resistant and susceptible *S. aureus* in response to cefoxitin (Figure 2) [35]. Microcalorimetry has been used for DST in mycobacteria. In the case of *M. avium*, it was applied to understand the effect of pre-incubation with ethambutol in potentiating the activity of streptomycin, and to distinguish each drug’s mechanism of action [36]. Microcalorimetry instruments work either with a liquid or solid medium (on slant tubes). Besides being advantageous for the study of mycobacterial strains that grow easiest on agar medium [34], the detection of mycobacterial growth by heat production is faster on a solid than a liquid medium [37]. Boillat-Blanco et al. [37] also showed that the MICs of four antibiotics against *M. abscessus* determined by microcalorimetry are in agreement with those obtained by microbroth dilution, within a range of two-fold dilutions. Importantly, microcalorimetry is capable of detecting resistance to clarithromycin in 72 h instead of the 14 days by microbroth dilution [37]. Using microcalorimetry, Howell et al. [34] accurately determined the MIC of three drugs against *M. smegmatis*, *M. avium*, and *M. tuberculosis*, and distinguished bacteriostatic from bactericidal effects by relating the bacterial growth rate with the duration of the lag phase. Besides speed, compared with gold-standard methods, microcalorimetry has the advantage of measuring bacterial growth in a continuous way without the consumption of additional reagents. If translated to high throughput, this technique can be of extreme value in the DST of slow-growing microorganisms.

## 4. Light-Based Methods

### 4.1. ATP Bioluminescence Assay

**Figure 3:**
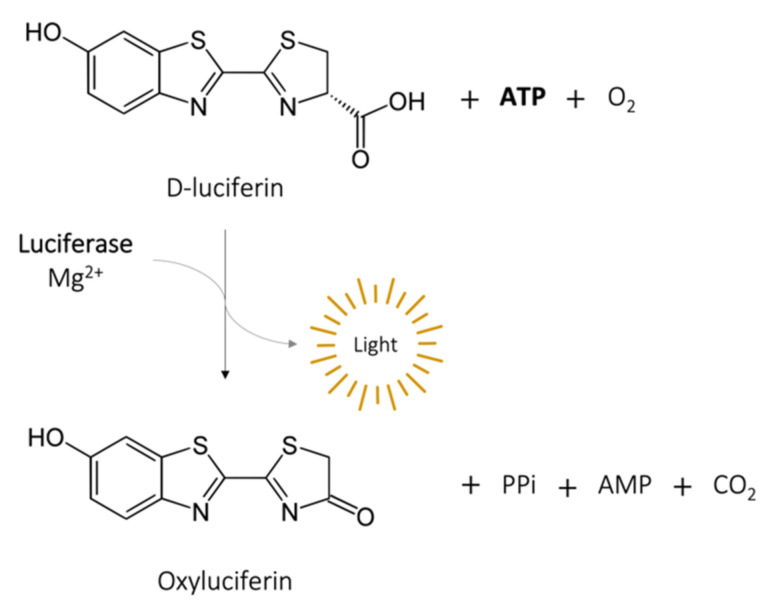
Bioluminescence reaction catalyzed by luciferase.

The measurement of adenosine triphosphate (ATP) produced by bacteria to determine cell viability, provides reliable DST results in a matter of hours, a great breakthrough from conventional methods [38,39]. The intracellular ATP in bacteria can be assessed by the ATP bioluminescence assay, which is based on the conversion of D-luciferin in oxyluciferin by luciferase in the presence of ATP and Mg^2+^, generating light [11] (Figure 3). The BacTiter-Glo microbial viability assay (Promega, Madison, WI) is a commercial kit with this principle. After incubation in the presence or not of the drug of interest, bacteria are mixed with a cocktail that contains a lysis buffer, luciferase, and D-luciferin. A luminescent signal will be produced, which is proportional to the intracellular ATP concentration and bacterial viability. It is recorded by a microtiter luminometer, or other instruments that measure luminescence and translated into relative light units (RLU). This method is particularly suitable for high-throughput assays. Cai et al. [40] tested six antibiotics alone and in combination against 100 strains of carbapenem-resistant *Acinetobacter baumannii*, *Pseudomonas aeruginosa*, and *Klebsiella pneumoniae* using the BacTiter-Glo assay and compared the results with a conventional CFU assay. Within 24 h, the bioluminescent assay identified inhibitory/non-inhibitory antibiotic combinations with an overall accuracy of 91%. Kapoor et al. [41] adapted the same assay for three rapid-growing mycobacteria (*M. immunogenum*, *M. chelonae*, and *M. abscessus*) and reported a linear relationship (r^2^ = 0.99) between intracellular ATP (RLU) and bacterial load (CFU/mL). Importantly, the BacTiter-Glo assay gives results in 1.5 h, compared to the 3 to 5 days required for these species by CFU assay. Using a similar luciferin–luciferase reagent, Beckers et al. [42] showed results for *M. tuberculosis* after 5 days of incubation instead of the 3 to 4 weeks required by a standard dilution method. Dong et al. [43] applied the BacTiter-Glo assay to personalized medicine using a microfluidic simulator that captures urinary tract pathogens from a urine sample with antibodies trapped on a membrane and measures ATP bioluminescence after antibiotic treatment. The specificity and rapidity of this method allowed the identification of the pathogens within 20 min and the evaluation of the antiseptic effects of eight antibiotics within 3 to 6 h.

### 4.2. Reporter Strains

**Figure 4:**
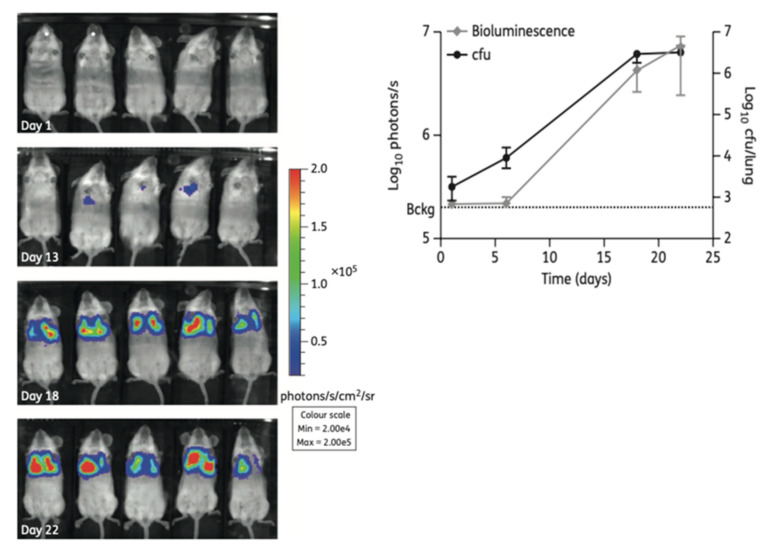
(**Left**): Non-invasive longitudinal monitoring of mice infected with a FFluc *M. tuberculosis* reporter strain after intraperitoneal injection of D-luciferin (IVIS^®^ Spectrum system). (**Right**): The bioluminescent signal obtained by live imaging correlates with CFU counting (from [47] under the terms of a CC BY-NC 3.0 license: https://creativecommons.org/licenses/by-nc/3.0/) (accessed on 8 December 2021).

ATP production can also be measured without the addition of any reagent, by genetically transforming bacterial strains to express the luciferase gene. Those reporter strains can either express the firefly luciferase (FFluc) gene, requiring the addition of D-luciferin to produce light, or the bacteria can be autoluminescent by expressing the whole luciferase operon (lux operon) [44]. Rocchetta et al. [45] transformed a clinical isolate of *Escherichia coli* with the lux operon and tested three antimicrobial agents measuring bioluminescence levels with an intensified charge-coupled device (ICCD) camera system and a microtiter luminometer. The antibiotics showed similar MIC and MBC by bioluminescence and by turbidity/CFU, independently of their mechanism of action or their bactericidal/bacteriostatic activity. A similar study was performed with *M. tuberculosis* strains expressing the FFluc or the lux operon [44]. The time to obtain MIC and MBC results was reduced to 3 and 6 days, respectively, and the values were comparable to those obtained by resazurin assay and CFU counting. Grant et al. [46] used a reporter strain with FFluc in a high-throughput assay to evaluate the activity of a series of compounds against replicative and also non-replicative *M. tuberculosis*, which is an important point in the case of this species. Another advantage of bioluminescent reporter strains is the easy application to DST in cell-based systems [44] and in animals by in vivo imaging [45,47] (Figure 4), which is extremely relevant and valuable for intracellular pathogens.

**Figure 5:**
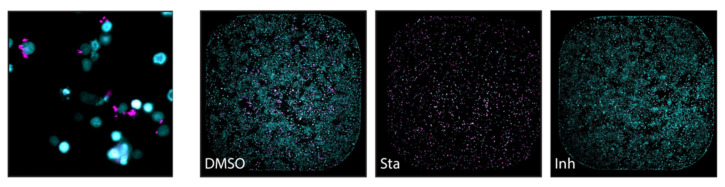
(**Left)**: Macrophages (nuclei stained with SYBR Green I–in cyan) infected with *M. tuberculosis* expressing a red fluorescent protein (DsRed) in magenta, analysed by high-content screening technology. (**Right**): The effect of treatments in macrophage and *M. tuberculosis* viability assessed by the same technique (entire well in one frame): both cells are viable in the presence of DMSO alone; most macrophages are killed by the cytotoxic compound staurosporine (Sta), and the viability of *M. tuberculosis* is significatively affected by the presence of the antimicrobial isoniazid (Inh) (from [52] under the terms of a CC BY 4.0 license: https://creativecommons.org/licenses/by/4.0/) (accessed on 8 December 2021).

Besides bioluminescence, bacterial strains can also be transformed to express fluorescent proteins [48]. Fluorescent reporter strains are widely used in DST given their ability to be imaged and quantified using several techniques, from plate readers to microscopy or flow cytometry. Like bioluminescence, fluorescence can be assessed in extra- and intracellular bacteria in vitro, and in in vivo imaging. Bioluminescence overcomes problems associated with in vivo fluorescence, such as poor penetration in live tissues or strong background signals, which also affects in vitro microtiter plate readings [48]. However, the large range of available colored fluorescent proteins that can be used in bacterial reporter strains and the advantage of not having to add an exogenous substrate makes it a valuable tool for in vitro high-throughput DST. Early et al. [49] used *M. tuberculosis* constitutively expressing red fluorescent proteins (mCherry or DsRed) to screen a library of 87,000 small compounds. Ollinger et al. [50] also screened a large compound set in 96- and 384-well plates using two red fluorescent protein-reporter strains of *M. tuberculosis*. A screen with the well-known antimycobacterial agent rifampicin showed that the reporter strains derive similar MIC values when the readout is in relative fluorescence units (RLU) or optical density, and the values were equivalent to the parental non-fluorescent strain. Fluorescence can be used as a marker for intracellular mycobacterial viability in a high-throughput manner, as shown by VanderVen et al. [51] in 384-well microtiter plates analysed in a fluorimeter, and by Manning et al. [52], using high-content microscopy to screen compounds against *M. tuberculosis* but also to assess their toxicity towards mammalian macrophages (Figure 5).

### 4.3. Metabolism-Based Light Methods

**Figure 6:**
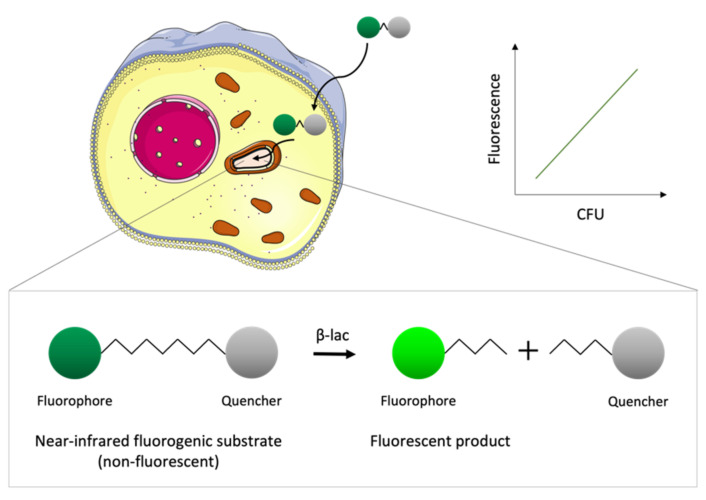
Schematic representation of the basis of the reporter enzyme fluorescence (REF) technology. The fluorogenic substrates cross the eukaryotic cell membrane and are taken up by intracellular bacteria that express the enzyme β-lactamase (β-lac). The substrate is not fluorescent due to the proximity of the fluorophore to a quencher; however, the product obtained upon hydrolysis by β-lac is fluorescent. The fluorescence of the product measured with a fluorimeter correlates with the number of viable bacteria inside the host cell.

Without having to express foreign genes in bacteria, which can affect their fitness, it is possible to use naturally occurring enzymes in bacteria as reporters, by the reporter enzyme fluorescence (REF) technology [48]. Kong et al. [53] developed near-infrared fluorogenic substrates of β-lactamase, better known as the main enzyme responsible for bacterial resistance against β-lactam antibiotics. This enzyme is not expressed in host cells but is present in a large variety of bacterial cells. When cleaved by β-lactamase, the appropriate substrates generate a fluorescent product that can be measured with a fluorimeter in in vitro setups, and in vivo using live animal imaging [53] (Figure 6). The fluorescent signal, resulting from the enzymatic activity of β-lactamase will be directly proportional to bacterial viability.

The BACTEC™ Mycobacteria Growth Indicator Tube (MGIT) system (BD Diagnostics) was developed to optimize the results obtained by mycobacterial reference methods such as the proportion method on LJ medium [9]. This automated system can be used to perform high-throughput DST qualitatively by growing mycobacteria in a tube with solid or liquid media containing an antibiotic. Embedded in silicone at the bottom of the tube there is an oxygen-quenched fluorochrome, which gets released as the oxygen is used by the bacteria’s metabolism. This assay is performed in equipment that handles almost a thousand tubes simultaneously and, according to the fluorescent signal emitted in each tube, indicates if the strain is resistant or susceptible to the antibiotic in comparison to a drug-free control tube. For *M. tuberculosis*, the MGIT system reduces the time to obtain final results from 42 days with the proportion method on LJ medium to 12 days [9]. The results obtained with the MGIT system are in close agreement with the reference methods for several conventional antibiotics [54,55,56] but, in some cases, are less specific and sensitive [57]. Although being efficient, the MGIT system still has the limitation of not being able to determine the MIC of the antibiotics, and not distinguishing between the growth of the study bacteria and possible contaminations.

## 5. Dynamic Models

**Figure 7:**
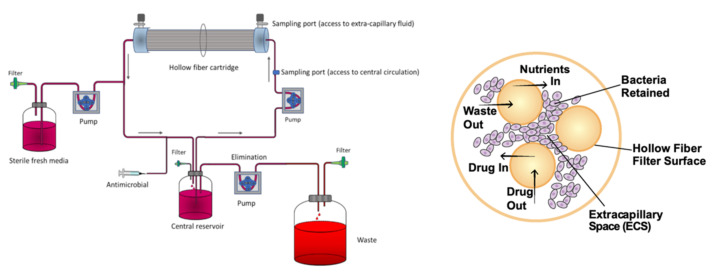
(**Left**): Schematic representation of a hollow fiber model system to study PK–PD properties of antimycobacterial compounds (from [62] under the terms of a CC BY 4.0 license: https://creativecommons.org/licenses/by/4.0/) (accessed on 8 December 2021). (**Right**): Cross-section of a hollow fiber cartridge, showing the bacteria trapped in the peripheral compartment, in contact with antibiotic and nutrients that can cross the fiber surface (from [63] under the terms of a CC BY 4.0 license: https://creativecommons.org/licenses/by/4.0/) (accessed on 8 December 2021).

In most in vitro DST studies, the activity of a drug is evaluated by exposing the bacteria to different drug concentrations in a static model, i.e., without monitoring dynamic factors such as nutrient concentration, pH variation, or oxygen availability [58]. Besides that, the MIC obtained using those conventional models does not take into consideration how the antimicrobial activity is affected by variations in drug concentration over time, as happens in vivo [59]. Dynamic models are used to study the pharmacokinetic–pharmacodynamic (PK–PD) aspects of a drug’s antimicrobial activity, by continuously refreshing the culture medium and clearing waste products and, at the same time, providing time-kill curves that reflect bacterial viability as a function of both time and drug concentration [58,59]. The hollow fiber model system (HFS) is the most common method to study PK–PD properties of antimycobacterial compounds (Figure 7). The HFS incorporates several semi-permeable hollow fibers that deliver and remove culture medium, oxygen, and, if it is the case, antibiotics, to a peripheral compartment where the mycobacteria are growing. The drug crosses the fiber and equilibrates rapidly within the compartment [58]. The drug kinetics are easily controlled, allowing exploration of the efficacy of novel compounds at different doses and drug combinations. Gumbo et al. [60,61] used an HFS with *M. tuberculosis* to study the relationship between the level of drug exposure, time, and sterilizing effects of anti-TB drugs [60], and to compare new drug regimens with conventional therapies [61]. In 2014, the European Medicines Agency (EMA) recommended the use of the HFS as a complementary method in the discovery of anti-tuberculosis drugs (procedure no. EMEA/H/SAB/049/1/QO/2014/SME). Although this in vitro model brings a very reasonable approximation to the effect a drug has on a patient, it is still a very expensive and time-consuming method. Additionally, it lacks important in vivo features such as immune cells, plasma proteins, and other compounds that interfere with the drug’s bioavailability [58,59].

**Figure 8:**
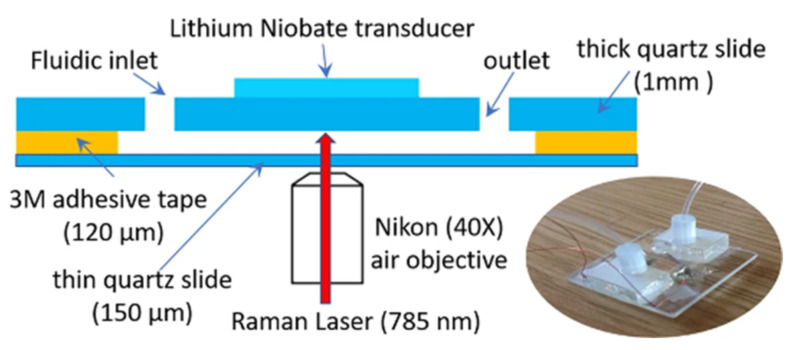
Schematic representation and photo of the acoustic trapping chamber designed by Baron et al. (from [68] under the terms of a CC BY 4.0 license: https://creativecommons.org/licenses/by/4.0/) (accessed on 8 December 2021).

A way of controlling the bacterial culture environment, mimicking in vivo conditions, and, at the same time, performing DST in a high-throughput manner, is by using a microfluidic system [64]. Cells are cultured in a chip composed of microchannels through which antibiotic dilutions circulate, and nutrients and oxygen are automatically renewed. The bacterial growth can be monitored for long periods and assessed, for example, by optical or spectroscopic methods [12]. There are several approaches to perform DST in microfluidic chips, from microfluidic agarose channels where bacteria are immobilized in contact with different drug conditions, enabling the MIC’s determination by plotting time-lapse images against the incubation time [65], to microfluidic channels filled with a pH-sensitive hydrogel that swells or shrinks in response to bacterial metabolism, measured in real time by Fourier transform reflective interferometric spectroscopy [66]. Both examples give MIC values from bacterial growth curves in 2 to 4 h for rapid-growing bacteria. Microfluidic chips are often used to study drug combination therapies. Lee et al. [67] developed a highly efficient mixing microfluidic chip to test the synergistic and antagonistic effects of different drug combinations against *S. aureus*, which was able to reduce detection time (from 24 to 4 h) and bacterial consumption (from 100 to 3 µL) in comparison to the broth microdilution technique. Baron et al. [68] used a microfluidic system to acoustically trap live *M. smegmatis*. Bacteria previously exposed to the antimycobacterial agent isoniazid levitated in a field produced by piezoelectric transducers, and their metabolic responses were monitored over time by Raman spectroscopy (Figure 8). The fact that the cells can be trapped in an acoustic field for long periods without losing viability [68] makes this technology very appealing to test the effect of drugs over time using a dynamic system.

## 6. In Vitro Biofilm Models

Biofilms are associations of microorganisms that tightly adhere to surfaces, enclosed by an extracellular matrix (ECM) of polysaccharides, DNA, and other molecules [69]. From aquatic surfaces to living tissues or medical devices, bacteria establish biofilms in the most diverse environments, which provides them protection against external aggressions, including antibiotics [70]. The special characteristics of the mycobacterial cell wall confer these species the ability not only to adhere to surfaces but also to form biofilms in air–media interfaces [71,72], making them prevalent in almost all environments. The different components of the biofilm ECM make it difficult for the penetration of antibiotics, create anaerobic areas where some compounds are not active, mainly because the bacteria are in a dormant form, and favor the development of antibiotic resistance by horizontal gene transfer [70].

In vitro DST with biofilm models can be performed in microtiter plates, where the bacteria are allowed to grow and adhere to the surface of the wells, and then be exposed to different concentrations of antibiotics. Bacterial viability within the biofilm or biofilm mass can be assessed using resazurin conversion or crystal violet staining, respectively [70]. Sanchez et al. [73] used the BacTiter-Glo viability assay to test three commercially available mouth rinses on a subgingival biofilm model of six different bacteria. Although no mycobacterial species were included in this work, these results show a correlation between bacterial viability by RLU and by colony counting, assuring ATP bioluminescence as a suitable method to assess drug efficacy in in vitro biofilm models.

**Figure 9:**
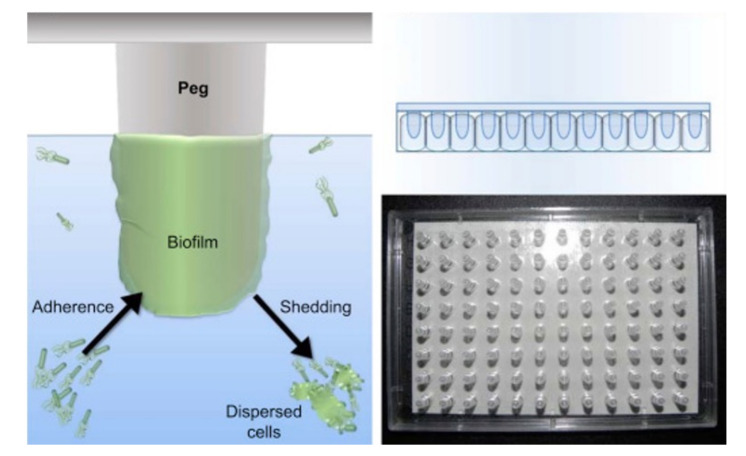
(**Left**): schematic representation of the MBEC Assay^®^, showing the bacteria on culture medium adhering to the peg on the lid of the plate and forming a biofilm. (**Right**): photo and representation of the lid containing 96 pegs, which fits any standard 96-well plate (from Innovotech^®^-http://www.innovotech.ca/) (accessed on 8 December 2021).

A variation of the biofilm formation method is the MBEC Assay^®^ (formerly Calgary biofilm device) (Figure 9) [74], an improved 96-well microtiter assay, where the biofilm grows in individual pegs placed above each well under batch conditions. The plate lid containing the pegs with the established biofilm can be transferred at different time-points to a new 96-well plate for drug testing.

**Figure 10:**
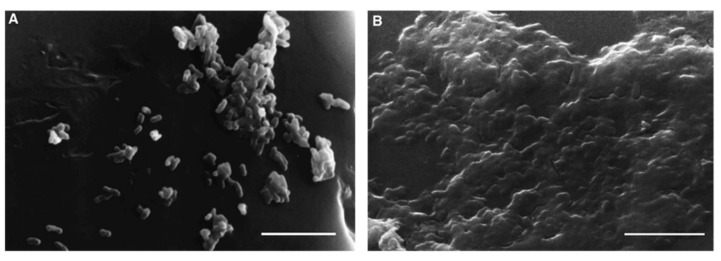
Images of scanning electron microscopy (SEM) of *M. phlei* growing on a peg after 3 days (**A**) and after 7 days (**B**), where a mature biofilm is visible. Scale bars = 5 μm (from [75], ©2001 Federation of European Microbiological Societies by permission of Oxford University Press).

Bardouniotis et al. [75] used an adaptation of the MBEC Assay^®^ to characterize biofilm growth of *M. phlei* by scanning electron microscopy (Figure 10) and testing the activity of known biocides against the biofilm. Rinsing and sonication of the pegs are required to detach the biofilms and then plate serial dilutions on agar plates to count CFUs. These methods are destructive and can only be applied to thin biofilms; thus, Solokhina et al. [76] used two non-invasive techniques to evaluate the metabolic activity of mature mycobacterial biofilms: isothermal microcalorimetry that measures the heat production of biofilms growing on solid surfaces; tunable diode laser absorption spectroscopy that measures biofilms’ production of oxygen and carbon dioxide. Both methods can be applied to DST, although in a high-cost and low-throughput manner.

**Figure 11:**
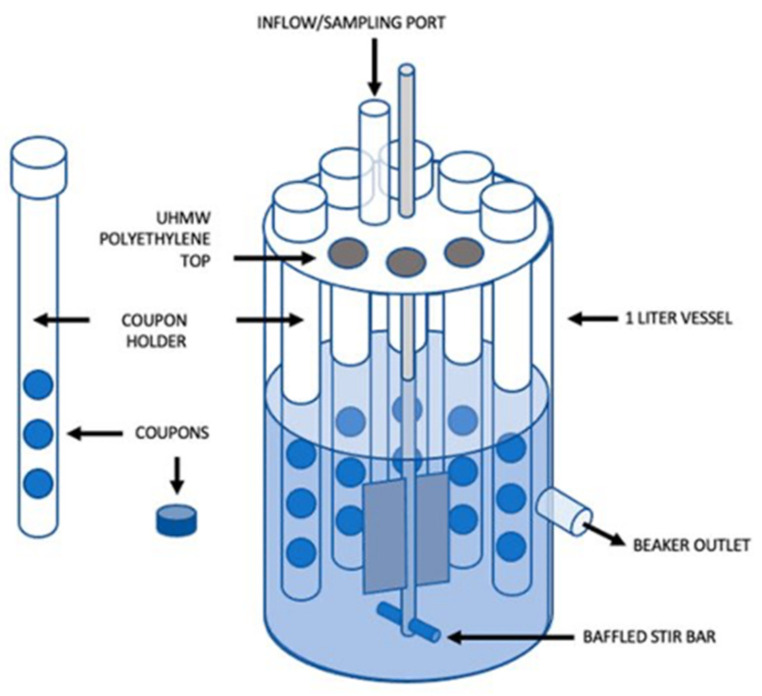
Schematic representation of a CDC Bioreactor for biofilm formation (from [80] under the terms of a CC BY 4.0 license: https://creativecommons.org/licenses/by/4.0/) (accessed on 8 December 2021).

All the previous models are, however, static, not reflecting the conditions experienced by natural biofilms. Open systems, in which parameters such as cell density, extracellular matrix, nutrients, gas, or metabolic products, can be modulated to better mimic the in vivo environment [77]. There are several dynamic biofilm systems [77]. One of the most common is the flow cell biofilm model, in which fluorescently-tagged bacteria form biofilms in the surface of microscope coverslips, connected to vessels that provide broth culture and antibiotic solution to the biofilm through a multichannel peristaltic pump. The biofilm can be monitored and its viability assessed by real-time non-destructive confocal laser scanning microscopy or by colony counting once it is removed from the flow cell [70]. Another way of growing biofilms in an open system is by using a CDC biofilm reactor (Figure 11). This commercially available device incorporates polypropylene holders, surrounded by culture media, which carry disk coupons. The system rotates, allowing the bacteria to adhere to the coupons and form biofilms. Flow speed and temperature can be controlled, and the coupons removed at any instance for analysis [70,77]. Armbruster et al. [78] used a CDC biofilm reactor to grow biofilms of four opportunistic bacteria found in potable water distribution systems, including *M. mucogenicum*, and test the biofilm susceptibility to a water disinfectant. As both previous methods are very time-consuming, Benoit et al. [79] developed a high-throughput system to screen flow biofilms, integrating microfluidic channels into 96-well microtiter plates.

Although extensively used to study different microorganisms, in vitro biofilm models are still not able to replicate the complex interaction between drug and bacteria in a natural biofilm, especially in a human tissue surface.

## 7. Three-Dimensional Models

### 7.1. In Vitro Granuloma Models

**Figure 12:**
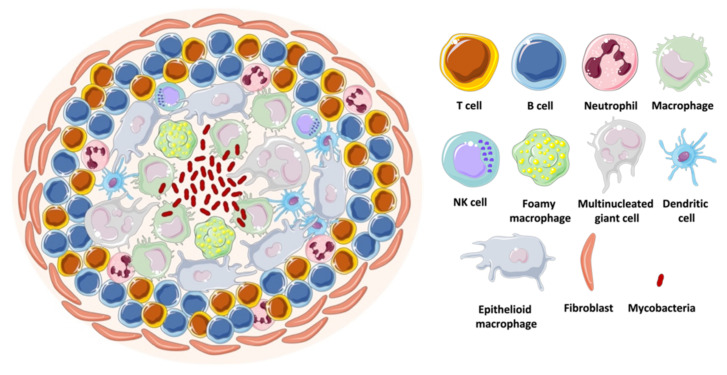
Schematic representation of a mycobacterial granuloma.

In vivo infection with mycobacteria is characterized by the formation of immune cellular organized aggregates called granulomas. These aggregates contain mainly specialized macrophages, such as high-lipid content foamy macrophages, large cytoplasm epithelioid macrophages, as well as multinucleated giant cells. This structure forms when mycobacteria are internalized by the host macrophages that start secreting cytokines and chemokines. Peripheral monocytes and T-lymphocytes are recruited to the site of infection, where the lymphocytes surround, together with a coat of fibroblasts and collagen, a core of differentiated macrophages, monocytes, neutrophils, dendritic cells, and intracellular or free mycobacteria [81,82] (Figure 12). The mycobacteria inside the hypoxic environment of the granuloma develop into a dormant state, characterized by the accumulation of intracytoplasmic lipid inclusions, loss of acid fastness, and resistance to the antibiotic rifampicin [81]. This bacterial phenotype is very difficult to replicate in vitro.

**Figure 13:**
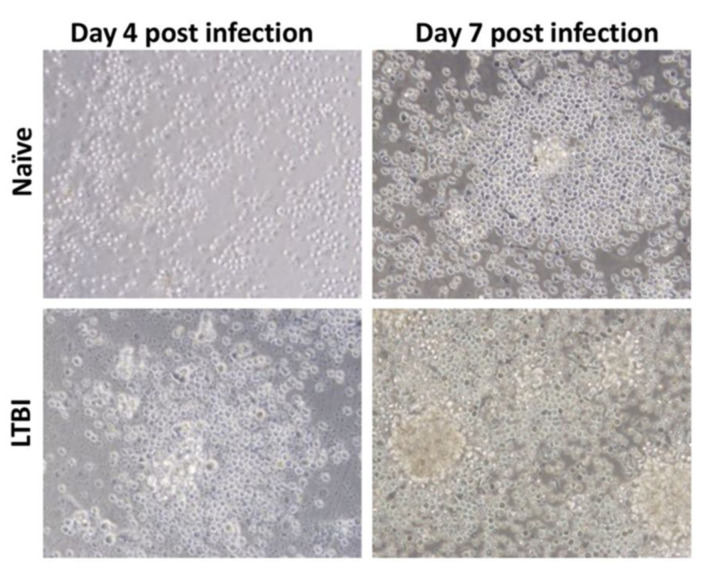
Light microscopy images (×40 magnification) of granuloma formation being influenced by the origin of the PBMCs. After infection with *M. tuberculosis*, PBMCs from individuals with latent TB aggregate into granulomas earlier than PBMCs from naïve individuals (from [93] under the terms of a CC BY-NC-SA 3.0 license: https://creativecommons.org/licenses/by-nc-sa/3.0/) (accessed on 8 December 2021).

The establishment of in vitro granuloma models is useful to test drug candidates against dormant and active mycobacteria. These enable DST at a reduced cost and increased control manner, in comparison to in vivo models. With the development of granulomas, the infection’s progression and cellular arrangement can be followed using light and/or fluorescent-based microscopy, and the number of spheroid structures, their size, and the number of bacteria can be quantified using appropriate software. A three-dimensional in vitro granuloma is achieved by infecting peripheral blood mononuclear cells (PBMCs) and culturing them in conditions that inhibit surface contact [81]. Several studies describe distinct approaches to construct in vitro granulomas using different supports/holders [83,84,85], extracellular matrixes (ECM) to accommodate the granuloma [86,87], levels of incorporation of immune cells [88,89], and mycobacterial species [81,90,91]. PBMCs can be collected from several animals. However, human PBMCs are more relevant since the pathology of mycobacterial infection, particularly the granuloma, is not easily replicated in other species [92]. These cells can even be isolated from TB-infected patients, as Guirado et al. [93] showed that there is a significant influence of immune memory on granuloma formation: PBMCs from latent TB-infected patients incubated with autologous serum and virulent *M. tuberculosis* develop into a granuloma-like structure, with the bacteria showing signs of latency earlier than when using PBMCs from TB-naïve individuals (Figure 13).

**Figure 14:**
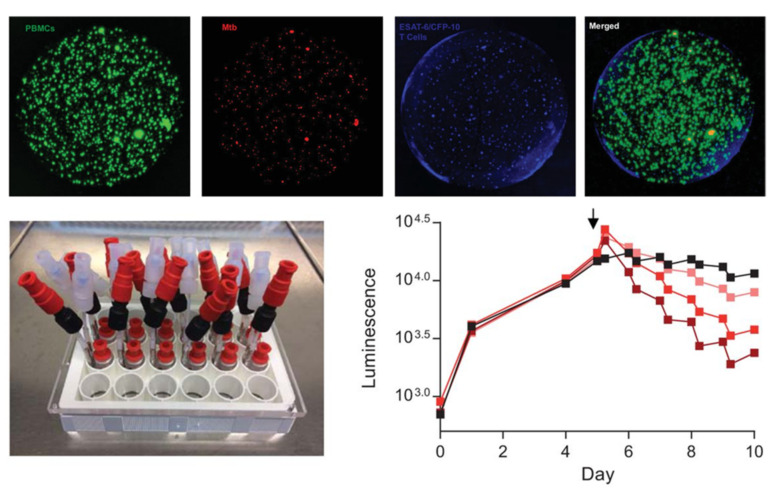
(**Top**): alginate–collagen microspheres with early-stage granulomas formed by human PBMCs infected with *M. tuberculosis* created by Bielecka and Tezera et al. PBMCs labelled with CellTrace CFSE (green); *M. tuberculosis* expressing a red fluorescent protein (mCherry); autologous ESAT-6 specific T cells labelled with CellTracker Blue. (**Bottom left**): Microfluidic system developed by the same group to study the pharmacokinetics of antibiotics against *M. tuberculosis*-infected granulomas inside microspheres. (**Bottom right**): Increasing concentrations of rifampicin (lighter to darked red, black represents a non-treated control), added at day 5 (arrow), accelerate the rate of *M. tuberculosis* death inside the granuloma, measured in a plate reader luminometer using a bioluminescent (lux) reporter strain (from [95] and [96] under the terms of a CC BY 4.0 license: https://creativecommons.org/licenses/by/4.0/) (accessed on 8 December 2021).

Although that study was performed in 24-well culture plates, the work with granulomas is scalable for high-throughput drug screening. Silva-Miranda et al. [94] assessed, with high-content screening technology, the activity of four antimycobacterial drugs in an in vitro granuloma model using human PBMCs infected with a fluorescent reporter strain of *M. tuberculosis*. The granulomas developed after three days in 384-well plates and the intragranuloma bacterial load was quantified by fluorescence reading with 3D analysis equipment. They observed significant differences in the MICs obtained from the granuloma model and those from extracellular bacterial assays, showing the limitations of conventional methods to mimic the different barriers a drug has to overcome to reach the bacteria in vivo. In a bioengineering approach, Bielecka and Tezera et al. [95,96] developed alginate–collagen microspheres incorporating *M. tuberculosis* fluorescent and bioluminescent reporters, human PBMCs, and ECM by bioelectrospray methodology (Figure 14, top). Granuloma-like structures developed inside the microspheres, closely mimicking the stress encountered by bacteria under in vivo conditions. Importantly, *M. tuberculosis* inside these structures is sensitive to pyrazinamide, as it happens in vivo, contrarily to what is observed in broth cultures. As the cells and bacteria are held within the microspheres, they adapted the model to microfluidic PK modelling with two input channels and one exit channel in a 24-well culture plate (Figure 14, bottom left). By adding the antibiotic rifampicin through the microfluidic system they showed that the killing of *M. tuberculosis* can be progressively accelerated by increasing the drug’s concentration through time (Figure 14, bottom right). This model can be refined by adjusting the microsphere composition to enable immune cell recruitment and create oxygen gradients, which better mimics the multiple microenvironments the mycobacteria experiences inside the host [97].

### 7.2. Organoids

In recent years, the need to find a new experimental model to overcome the limitations presented by in vitro assays with isolated cells and the difficulties imposed by animal models that fail to replicate the complexity of human diseases led to the development of organoids. Organoids are three-dimensional structures formed by a diversity of cells that self-organize into a small-scale organ, with similar tissue organization, immune response, and physiological characteristics to an in vivo organ [98]. Starting from human embryonic or induced pluripotent stem cells, or organ-specific adult stem cells, it is possible to grow in vitro organoids that mimic most human tissues, from retinal, cerebral, or hippocampal to liver, intestinal, or lung organoids [98]. Potential medical applications of organoids are vast, including in the field of host–pathogen interactions. Experimental studies were published of infections by *Shigella*, *E. coli*, *Clostridium difficile*, or *Salmonella* Typhi in intestinal organoids, or *Helicobacter pylori* in gastric organoids [99]. Several approaches have been described to develop lung organoids [100], and even apply them to high-throughput screening of compounds. For example, Danahay et al. [101] grew lung organoids derived from primary human airway basal cells, the so-called bronchospheres, in 384-well plates and screened 5000 different secreted proteins to identify which could influence basal cell differentiation into goblet cells, the lung cells responsible to produce mucus. In a pioneering way, Han et al. [102] used lung organoids derived from human pluripotent stem cells infected with SARS-CoV-2 to identify antiviral compounds among a library of FDA approved drugs, including imatinib, a drug used in clinical trials to treat COVID-19 patients.

**Figure 15:**
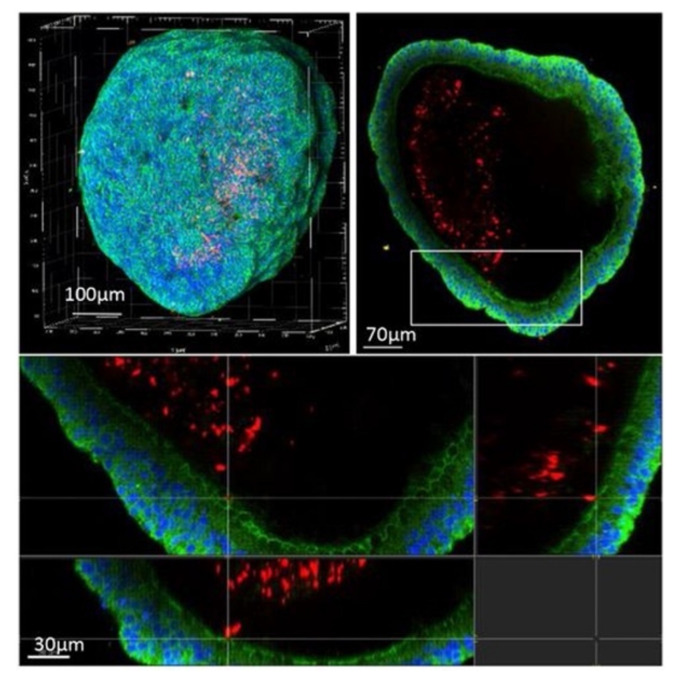
Confocal microscopy of an airway organoid infected with *M. tuberculosis* expressing a red fluorescent protein (DsRed) 4 days post-infection. Nuclei are labelled with DAPI (blue) and cellular membranes with CellMask green (green) (from [103] under the terms of a CC BY-NC-ND 4.0 license: https://creativecommons.org/licenses/by-nc-nd/4.0/) (accessed on 8 December 2021).

Studies of organoid infection with mycobacteria are scarce. A preprint study reported successful microinjection of fluorescent *M. tuberculosis* into human airway organoids [103] but did not perform DST. They observed, by confocal microscopy, mycobacteria in the lumen of the organoid interacting with epithelial cells (Figure 15); dissociation of the organoid and CFU counting showed decreased bacterial load after a week of infection but recovery by week 3 post-infection. RT-qPCR analysis of the organoids showed induced expression of cytokine and antimicrobial peptide genes upon *M. tuberculosis* infection. It is of extreme value to invest in organoid science as a new approach to finding new treatments for infectious diseases. It is by now the closest we get to simulate human disease without depending on animal testing, which is laborious, expensive, requires a high level of expertise, inevitably raises ethical issues, and often has a poor correlation with clinical outcomes [104]. However, and particularly with lung organoid models, there are still some barriers to get by, such as the introduction of immune cells and vasculature. The vascularization of the hydrogels that sustain the 3D structure of the organoid culture [105] could be a solution to the latter.

**Figure 16:**
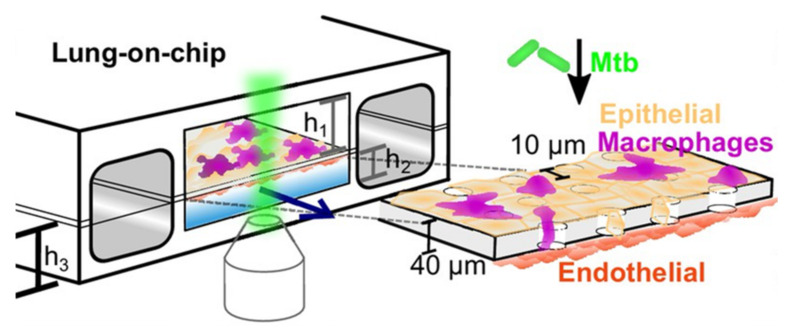
Schematic representation of the murine ALI lung-on-a-chip model with *M. tuberculosis* (green) infection adapted to microscopy imaging, established by Thacker et al. The upper compartment with air-exposed epithelial cells (yellow) represents the alveolar region of the lung and the lower compartment with endothelial cells (red) in contact with culture medium represents the vascular region. Fluorescent-tagged macrophages (magenta) can migrate through the porous membrane that separates the compartments. h_1_ = 1 mm, h_2_ = 250 mm, h_3_ = 800 mm (from [107] under the terms of a CC BY 4.0 license: https://creativecommons.org/licenses/by/4.0/) (accessed on 8 December 2021).

The association of organoid models with microfluidic systems resulted in the technology of organ-on-a-chip, a platform suitable for high-throughput drug screening [64]. Many types of organs have been adapted to chips, including the lung. As in simple lung organoids, the establishment of an air–liquid interface (ALI) is essential to expose the cells at their apical side to the air, while being in contact with a nutrient- and growth factor-rich medium on their basal side [106]. Thacker et al. [107] established a murine ALI lung-on-a-chip model with *M. tuberculosis* infection (Figure 16). The chip is constituted by two compartments separated by a porous membrane: the upper one in contact with the air and populated by alveolar epithelial cells; the lower one with endothelial cells in contact with liquid medium. Fluorescent macrophages are also present and can migrate between the two compartments. After infection of the cells in the epithelial side with *M. tuberculosis*, the dynamics of the infection were assessed by time-lapse microscopy. The development of a lung-on-a-chip model of *M. tuberculosis* infection with human cells would be the next step forward in the challenging path to have an in vitro model that closely predicts a clinical outcome. As mycobacteria also populate other organs, it would be interesting to test its infection on other chips, such as skin-on-a-chip [108], to study, for example, *M. abscessus* infection; or even on multi-organs-on-a-chip, a recent breakthrough also known as human-on-a-chip, in which cells of different organs are connected by microchannels that simulate blood vessels in a single chip [109].

## 8. Final Considerations

The development of new antimicrobial agents strongly depends on whether their activity in vitro translates, in a first instance, to a similar activity in vivo and then, in a later phase of the drug discovery process, to clinical effectiveness. For that, there is a need to test antimicrobial compounds in in vitro setups as accurately as possible in simulating the in vivo complexity of infection and, at the same time, on a scale big enough to respond to the urge for new molecules to face drug resistance issues.

Conventional methods such as broth or agar dilution, or even the different agar diffusion techniques, are reliable for aerobic rapid-growing bacteria but require a lot of manual work and the results take a long time to obtain. For slow-growing bacteria, e.g., many species of mycobacteria, it is not even possible to perform a disk diffusion test or an E-test, as the effect of the antibiotic is lost before the bacterial culture reaches a growth point in solid media that can be visible. Moreover, the fact that these traditional methods rely mostly on qualitative observation to obtain quantitative results is one of their drawbacks.

Microcalorimetry gives a step forward in the monitorization of bacterial growth in comparison with previous methods, as it enables assessment in real time of the response of the bacteria to the drug without having to add any reagents. It solves the issue of reading inconstancies of optical methods, as it measures an inherent product of bacterial metabolism and growth, heat, and, at the same time, facilitates the study of slow-growing microorganisms. Thus, microcalorimetry is a good technique to perform DST with mycobacteria; however, the equipment is expensive and does not yet allow high-throughput drug screening. That can be achieved by a microfluidic system, which has also the advantage of automatically renewing the culture media, closely mimicking the exchanges of nutrients and toxic waste that happens in vivo. The kinetics of drug delivery is controlled, with a lot of information on drug activity being gathered. These dynamic systems operate on a micro-scale, such as on chips, but also on a macro-scale, such as the hollow fiber system, which is frequently used to study mycobacterial susceptibility. Nonetheless, these models are costly and lack important features of the in vivo environment, such as the interference of host cells and proteins.

Mycobacteria can persist in an environment for long periods due to their ability to easily attach to surfaces and form biofilms. Biofilm formation is a concern not only in natural environments but also in a hospital context, as medical devices such as catheters and ventilation and water systems are prone to bacterial establishment and dissemination. Inside an organism, bacteria settle as biofilms in several tissues, hampering the activity of antibiotics. In DST, it is, then, essential to consider the ability of the drug to surpass the barriers conferred by the biofilm. Traditional bacterial culture tools such as microtiter plates can be adapted to study in vitro biofilm formation and test antibiotic activity. The addition of microfluidic systems and reporter bacteria improves the throughput and the accuracy to environmental biofilms, however, in vitro studies with biofilms on animal or human tissue are lacking from most host–pathogen interaction research.

In fact, the current standardized methods to perform DST analyze the effect of the antibiotic against bacteria in the planktonic state, not taking into consideration all the barriers the drug must surpass in vivo, mainly in the case of intracellular bacteria. A drug can show activity when tested directly against the pathogen, but the effect can be lost if the compound does not have access to the bacteria inside the host cells. The situation can happen the other way around, as some drugs are known to facilitate the action of other drugs, for example, by altering the permeability of the host cell membrane and allowing the other to reach the bacteria, but do not show direct activity by themselves. In vitro assays with infection of eukaryotic cells to evaluate drug susceptibility are performed routinely by groups that study intracellular bacteria. For mycobacteria, the most common procedure is to infect in vitro cultures of macrophages. These cells can be obtained from primary cultures of murine or human precursors or different cell lines, such as RAW 264.7 murine macrophage cells, THP-1 human monocyte cell line, or A549 human alveolar epithelial cell line; however, infection of human PBMCs is more accurate for the study of human diseases. These methods are standardized and give insightful information before testing in animal models, such as the toxicity of new drugs to the host cells. Nevertheless, they do not mimic all in vivo aspects. A hallmark of mycobacterial infection is the formation of granulomas, where mycobacteria are enclosed sometimes in a dormant state. In vitro granuloma models allow studying of these pathogens in an environment very close to what they experience inside the host. DST performed in in vitro granulomas indicates whether the drug can pass through the several layers of host cells and have activity against replicative or non-replicative bacteria, which is a great breakthrough in comparison with conventional in vitro techniques mentioned above.

In the last decade, as scientific knowledge tried to recreate, in vitro, a model as close as possible to an in vivo organ, organoids came into the picture. Taking advantage of the ability of stem cells to differentiate into any type of adult cell, it is now possible to culture tissue-specific human cells that assemble into a 3D structure resembling a human organ. High-throughput DST can be achieved by the technology of organs-on-a-chip, where organoids are associated with microfluidic systems. The use of in vitro granuloma or organoid models avoids testing new compounds in animals in an early stage of drug development, reducing costs and contributing to animal welfare in scientific research.

Overall, the readout of granuloma and organoid models can be facilitated by infection with reporter bacterial strains, either expressing a bioluminescent or a fluorescent gene. The in vitro screening is easily performed with an ICCD camera that measures bioluminescence levels, fluorescence microscopy, or flow cytometry to image fluorescent bacteria, or using a simple plate reader that can measure both. Indeed, it is advantageous to transform bacteria to express both fluorescence and bioluminescence. Fluorescence is very practical, as there is a wide range of fluorescent proteins available and there is no need to add any substrates, however, the imaging often suffers from background issues. Bioluminescence overcomes that problem due to the specificity of the substrate that is added, or because the strain can be autoluminescent, with no need for adding a reagent. Therefore, recombinant bacteria expressing fluorescent and bioluminescent genes can be used as markers for bacterial load and for following the progress of the infection, either in vitro, or also live on animals, in a low-cost and non-invasive way.

In conclusion, conventional single-cell methods are useful in a clinical context to determine the drug susceptibility of a pathogen isolated from patients, as well as a preliminary approach to assessing the susceptibility of a microorganism to a new compound. However, a high-throughput technique should be considered to screen a high number of compounds rapidly and reliably. Compounds that show activity against planktonic bacteria can then be tested using models that mimic an in vivo environment, such as biofilms or in vitro granulomas, specifically for mycobacteria, or organoids, that can be coupled to microfluidic systems. These more complex models are important tools to characterize a restricted number of potential drugs in more advanced stages of drug discovery programs (Table 1).

## Figures and Tables

**Table 1:** Summary comparison table of in vitro DST methods.

		Structure	Throughput	Correlation to In Vivo	MIC/MBC ^a^	Drug Combinations ^a^	Time ^b^	Resources ^c^	Suitability for Slow Growers	Real-Time Monitoring	Requires Genetically Altered Bacteria	Drug kinetics Monitoring	Non-Replicative Bacteria
**Dilution methods**													
	Broth dilution	2D	Low	Low	✓	✓	++	++	✓	✗	✗	✗	✗
	Agar dilution	2D	Low	Low	✓	✗	++	++	✓	✗	✗	✗	✗
**Agar diffusion methods**													
	Kirby–Bauer	2D	Low	Low	✗	✓	++	++	✗	✗	✗	✗	✗
	E-test	2D	Low	Low	✓	✓	++	++	✗	✗	✗	✗	✗
**Microcalorimetry**		2D	Low	Medium	✓	✓	+	+++	✓	✓	✗	✗	✗
**Bioluminescence assays**													
	BacTiter-Glo viability assay	2D	High	Medium	✓	✓	-	++	✓	✓	✗	✗	✓
	Bioluminescent reporters ^1^												
	Expressing FFluc	2D	High	Medium	✓	✓	-	++	✓	✓	✓	✗	✓
	Expressing lux operon	2D	High	Medium	✓	✓	-	+	✓	✓	✓	✗	✓
**Fluorescence reporters ^1^**													
	Expressing fluor. proteins	2D	High	Medium	✓	✓	-	+	✓	✓	✓	✗	✗
	Metabolism-based light meth.	2D	High	Medium	✓	✓	-	+	✓	✓	✗	✗	✗
**Dynamic models**													
	Hollow fibber model system	2D	Medium	High	✓	✓	++	+++	✓	✗	✗	✓	✗
	Microfluidic chips	2D	High	High	✓	✓	-	++	✓	✓	✗	✓	✗
**Biofilm models**													
	MBEC Assay^®^	2D	Medium	Low	✓	✓	++	++	✓	✓	✗	✗	✗
	Dynamic biofilm models	2D	Low/med.	Medium	✓	✓	++	+++	✓	✓	✗	✓	✗
**In vitro granuloma models**													
	Multicellular lung tissue	3D	Low/med.	High	✓	✓	+++	+++	✓	✓ ^2^	✗	✗	✓
	PBMC-based granuloma	3D	Med./high	Medium	✓	✓	+++	+++	✓	✓ ^2^	✗	✗	✓
	Bioelectrospray 3D model	3D	Medium	High	✓	✓	+++	+++	✓	✓ ^2^	✗	✓	✓
**Organoids**													
	Lung organoids	3D	Low/med.	High	✓	✓	+++	+++	✓	✓ ^2^	✗	✗	NT
	Organ-on-a-chip	2D	High	High	✓	✓	+++	+++	✓	✓	✓	✓	NT

^a^ “✓” indicates that the parameter applies to the method and “✗” indicates that the parameter does not apply to the method. ^b^ Time refers to the period needed to establish the model and obtain results, in which “-” is the shortest period and “+++” is the longest. ^c^ Resources concerns the cost in reagents, material, and equipment, in which “+” is the cheapest and “+++” is the most expensive method. “++” indicates a intermediate condition between “+” and “+++”. ^1^ The bioluminescent and fluorescent reporter bacterial strains can be used alone to assess the direct antimicrobial activity of a compound. They can also be incorporated into other models, two-dimensional (2D) or three-dimensional (3D), to monitor the infection over time. ^2^ The infection can be monitored in real time if a reporter strain is included. NT–not-tested yet for the model.
